# Journalistic Notes

**Published:** 1891-11

**Authors:** 


### Journalistic Notes.

Journal of Balneology and Dietary.—We take pleasure in advising our readers that A. N. Bell, A. M., M. D., of Brooklyn, Frank Woodbury, A. M., M. D., of Philadelphia, and Geo. H. Rohé, M. D., of Catonsville, Md., will in future conjointly direct the editorial department of this *Journal.* The well-earned reputation of these gentlemen is sufficient assurance that they will discuss with authority the subjects coming within the scope of this publication. Drs. Bell and Rohé have long been prominently identified with the progress of sanitary science in this country, and as authors of important works on climatology, balneology, and hygiene. Dr. Woodbury is too well-known as an accomplished medical editor and writer on dietetic subjects to require further introduction. Extensive arrangements have been made for contributors among the most noted experts in this country and abroad, who are regarded as authorities upon the special subject to which this magazine is devoted. Address, P. O. Box 1670, New York.

---

The October number of the *Alienist and Neurologist* contained one of the most complete and practical papers on the subject of Traumatic Neuroses and Spinal Concussion ever written; the best paper extant on The Insanity of Torquato Tasso; an Exhaustive

Study of Criminals and their Cranial Development; The Weight of the Brains of the Feeble-Minded; A Study of the Heredity of Inebriety. The respective writers are Guiseppe Seppilli, Italy; W. W. Ireland, Scotland; G. Frank Lydston, Chicago; A. W. Wilmarth, Pennsylvania; and T. L. Wright, Ohio. It also contained the usual selections, editorials, hospital notes, reviews, etc. The editor is C. H. Hughes, M. D., 500 N. Jefferson avenue, St. Louis. Subscription, $5.00 per annum; single copy, $1.50.

---

*The Daily Medical News* is a new venture in medical periodical literature. It is edited and published in Philadelphia by Dr. Joseph F. Edwards. The third number, dated October 13, 1891, has been sent us, and we commend the enterprise that puts forth such a daring scheme as the publishing of a daily medical newspaper.

---

"Do you rectify mistakes here?" asked a gentleman, as he stepped into a drug store. "Yes, sir, we do, if the patient is still alive," replied the urbane clerk.—*Ex.*

